# Characterization and genetic dissection of resistance to spotted alfalfa aphid (*Therioaphis trifolii*) in *Medicago truncatula*

**DOI:** 10.1093/jxb/ert305

**Published:** 2013-09-21

**Authors:** Lars G. Kamphuis, Judith Lichtenzveig, Kefan Peng, Su-Min Guo, John P. Klingler, Kadambot H. M. Siddique, Ling-Ling Gao, Karam B. Singh

**Affiliations:** ^1^CSIRO Plant Industry, Private Bag 5, Wembley, WA 6913, Australia; ^2^The UWA Institute of Agriculture, University of Western Australia, Crawley, WA 6009, Australia; ^3^ Current address: Department of Environment and Agriculture, Curtin University, Bentley, WA 6102, Australia; ^4^Key Laboratory of Genetics & Biotechnology, Ministry of Education, Nanjing Forestry University, Nanjing 210037, PR China

**Keywords:** Antibiosis, antixenosis, EPG, herbivory, phloem, quantitative trait locus (QTL), sap-sucking insect, vein chlorosis.

## Abstract

Aphids cause significant yield losses in agricultural crops worldwide. *Medicago truncatula*, a model legume, cultivated pasture species in Australia and close relative of alfalfa (*Medicago sativa*), was used to study the defence response against *Therioaphis trifolii* f. *maculate* [spotted alfalfa aphid (SAA)]. Aphid performance and plant damage were compared among three accessions. A20 is highly susceptible, A17 has moderate resistance, and Jester is strongly resistant. Subsequent analyses using A17 and A20, reciprocal F_1_s and an A17×A20 recombinant inbred line (RIL) population revealed that this moderate resistance is phloem mediated and involves antibiosis and tolerance but not antixenosis. Electrical penetration graph analysis also identified a novel waveform termed extended potential drop, which occurred following SAA infestation of *M. truncatula.* Genetic dissection using the RIL population revealed three quantitative trait loci on chromosomes 3, 6, and 7 involved in distinct modes of aphid defence including antibiosis and tolerance. An antibiosis locus resides on linkage group 3 (LG3) and is derived from A17, whereas a plant tolerance and antibiosis locus resides on LG6 and is derived from A20, which exhibits strong temporary tolerance. The loci identified reside in regions harbouring classical resistance genes, and introgression of these loci in current medic cultivars may help provide durable resistance to SAA, while elucidation of their molecular mechanisms may provide valuable insight into other aphid–plant interactions.

## Introduction

Aphids, the largest group of sap-sucking pests, are in the superfamily Aphidoidea in the homopterous division of the order Hemiptera. They cause damage to their host plants directly by modifying plant metabolism and ingesting plant nutrients from the phloem and indirectly by vectoring plant-pathogenic viruses. Control of aphids using insecticides has proven difficult as aphids often develop insecticide resistance. Thus, durable genetic resistance to aphids in agricultural crops offers a sustainable means of combating these pests. Resistance to sap-sucking insects is often controlled by dominant or co-dominant plant genes that may be insect genotype specific. This specificity has suggested a gene-for-gene model reminiscent of classical resistance (*R*) gene-mediated defence against plant pathogens. Cloning of the tomato *Mi-1* gene against a root-knot nematode and against specific isolates of the potato aphid (*Macrosiphum euphorbiae*) (Rossi *et al.*, 1998) confirmed the relevance of the gene-for-gene model to plant–aphid interactions. Through products of *R* genes, host plants may recognize specific, aphid-derived molecules (e.g. effectors) and mount a defence response against the insect. Resistance to aphids involves a variety of resistance mechanisms (Smith, 2005), including maintenance of plant growth and seed production despite aphid infestation (tolerance), reduction in aphid preference (antixenosis), or repression of aphid growth and development (antibiosis). Host resistance to aphids when deployed in monocultured crops can be overcome by newly evolved aphid biotypes as seen, for example, in lettuce, melon, soybean, and wheat (McCreight, 2008; Chen *et al.*, 2009; Randolph *et al.*, 2009; Dogimont *et al.*, 2010; Michel *et al.*, 2010). The use of a combination of major genes and/or quantitative basal resistance loci could provide a practical strategy to solve the problem of resistance breakdown by new biotypes (Palloix *et al.*, 2009).

Spotted alfalfa aphid (SAA; *Therioaphis trifolii* f. *maculata* Buckton) was first found on alfalfa in New Mexico, USA, in 1954 (Lloyd *et al.*, 1983) and first seen in Australia in 1977 (Lake, 1989). SAA is a cosmopolitan pest of legumes mainly in the tribes Trifoliae and Loteae (Blackman and Eastop, 2000). SAA is a major insect pest of alfalfa (*Medicago sativa* L.) as well as annual medic (*Medicago*) species and can cause severe and widespread damage to subterranean clover (*Trifolium subterraneum*) (Lake, 1989). SAA severely inhibits seedling establishment and plant growth, affecting the quality and quantity of herbage produced, particularly hay, with an estimated 25% loss in production (He and Zhang, 2006). This aphid species has developed biotypes that have overcome resistant alfalfa varieties (Nielson *et al.*, 1970) and has a close relative, the spotted clover aphid (*Therioaphis trifolii* Monell), which is a genetically distinct form of the same species (*T. trifolii*) distinguished by their ability to survive successfully on red clover (*Trifolium pratense* L.) and alfalfa (Sunnucks *et al.*, 1997; Milne, 1998).

SAA’s hallmark of plant damage on susceptible plants is systemic vein chlorosis (VC). In the model legume *Medicago truncatula* (barrel medic), SAA-induced VC can occur exclusively on expanding systemic leaves approximately orthostichous to infested leaves (Klingler *et al.*, 2007). The incidence and patterns of VC suggest that it occurs where leaves are still sinks for photoassimilates rather than sources. Constituents of SAA saliva are transported over several internodes within alfalfa (Madhusudhan and Miles, 1998). Therefore, systemic VC is probably a host response to a bioactive component(s) or derivative(s) of SAA saliva. In *M. truncatula*, the phenotype can be correlated with host susceptibility to SAA (Klingler *et al.*, 2007).

Other aphid species recorded on legumes include: bluegreen aphid (BGA; *Acyrthosiphon kondoi* Shinji), pea aphid (PA; *Acyrthosiphon pisum* Harris), cowpea aphid (*Aphis craccivora*), and green peach aphid (*Myzus persicae* Sulzer). In recent years, resistance to most of these aphid species has been identified and characterized in *M. truncatula* (Klingler *et al.*, 2005, 2007, 2009; Gao *et al.*, 2007*a*,*b*, 2008, 2010; Guo *et al.*, 2009, 2012; Stewart *et al.*, 2009; Kamphuis *et al.*, 2012). Effective resistance against the pasture legume specialists BGA and SAA has successfully been introgressed into commercial cultivars of *M. truncatula* (Lake, 1993*a*,*b*, Hill, 2000; Gao *et al.*, 2007*b*), and, coincidentally, resistance to PA was introduced in one particular cultivar, Jester (Guo *et al.*, 2009). Resistance to BGA, PA, and SAA in Jester is controlled by distinct single dominant genes identified using mapping populations derived from the closely related line Jemalong A17 (~89% identical to Jester) or line A20 (highly susceptible to all three aphids) (Klingler *et al.*, 2005; 2007; Gao *et al.*, 2008; Guo *et al.*, 2009). Distinct quantitative trait loci (QTLs) affecting antibiosis and tolerance to BGA and PA have also been identified in *M. truncatula* line Jemalong A17 compared with A20, where antibiosis traits against both aphid species are controlled by the same locus, albeit with different degrees of resistance, and by two independent loci for plant tolerance (Guo *et al.*, 2012).

In *M. truncatula*, two different phenotypes of resistance to SAA have been identified (Gao *et al.*, 2007*b*). The resistance phenotype observed in *M. truncatula* Jester (as well as in cultivars Cyprus and Caliph) is highly effective, where all nymphs die within 24–48h, regardless of the developmental stage of the aphids (Gao *et al.*, 2007*b*). A second, less-effective resistance phenotype was observed in *M. truncatula* A17 (and in cultivar Mogul compared with cultivar Borung and accession A20), where SAA mortality rates were moderate (40–80%) and growth rates were suppressed. This raised the question of whether the two resistance phenotypes are controlled by independent genes or the same gene in different genetic backgrounds. It was demonstrated that SAA resistance in *M. truncatula* Jester was controlled by a major dominant gene termed *TTR* (for *Therioaphis trifollii* resistance) (Klingler *et al.*, 2007). Jester is also likely to contain components of the SAA resistance observed in its recurrent parental accession A17. Therefore, in the current study, we characterized the moderate resistance to SAA in the *M. truncatula* line A17 and compared it with the highly susceptible *M. truncatula* line A20 and the strongly resistant *M. truncatula* line Jester, using both choice and non-choice tests, and the electrical penetration graph (EPG) technique. We found that the moderate SAA resistance in A17 involved both antibiosis and tolerance, and that resistance was phloem based. QTL analysis using a recombinant inbred line (RIL) population from a cross between A17 and A20 revealed a distinct QTL for SAA antibiosis derived from A17 and another plant tolerance and antibiosis locus derived from A20. A third QTL acting late in response to SAA infestation (21 d post-infestation, dpi) was also identified.

## Materials and methods

### Plants and aphids

Three accessions were used in this study: A17, a derivative of the cultivar ‘Jemalong’ and the reference accession for the *M. truncatula* species, ‘Jester’ a near isogenic line derived from A17, and A20. To ensure uniform germination, seeds were scarified using sandpaper, transferred to a Petri dish lined with blotting paper, and irrigated with sterile water. The seeds were kept at room temperature for 48h; germinated seedlings were planted in *Arabidopsis* mix (Richgro Garden Products, Jandakot, Western Australia 6164). Plant growth conditions were as described previously by Klingler *et al.* (2009).

The SAAs used in this study were an asexual, parthenogenetic strain collected in Western Australia, derived from single-aphid isolates, and maintained in the laboratory as described by Gao *et al.* (2007*b*).

### Statistical analyses

Statistical analyses were done using JMP v.9 (SAS Institute, Cary, NC, USA). Data frequency distribution was tested for normality using the Shapiro–Wilk test. Observations with frequency distributions significantly (*P*<0.05) different from normal were mathematically transformed to conform more closely to the analysis of variance (ANOVA) assumptions. The arcsine transformation was used to normalize proportion data; for other data, transformation formulae were determined empirically (Kuehl, 2000). In figures and tables, the untransformed values are presented for simplification. Transformed/untransformed data were evaluated for homogeneity of variances using Bartlett’s test; Welch *F*-tests (or *t*-tests for unequal variances) were performed when heteroscedasticity of variances was found. The specific factorial models employed in ANOVA are presented for each experiment. Post-hoc multiple comparisons of means were done using Tukey–Kramer HSD tests (α=0.05).

### Aphid performance in choice and non-choice experiments

Two-week-old plants were infested with two apterous adults of SAA in a growth chamber and evaluated in two independent experiments in the glasshouse. The ‘non-caged’ experiment, where aphids were free to move among accessions, was set in a split-plot design with treatment (infested or non-infested) as the main plot and accessions (Jester, A17, and A20) as the secondary plot. The ‘caged’ experiment, with each plant enclosed in an open-air cage following infestation and for the duration of the trial, was set in a completely randomized design. For each experiment, there were six to eight experimental units. Seventeen days after infestation, the aphids on each plant were gently brushed off and counted to determine aphid number (AN). The following plant measurements were also taken: shoot fresh weight (SFW), total number of leaves (TL) and the proportion of leaves showing VC (VC/TL). Data was analysed as non-transformed (SWF) or following mathematical transformation, as the arcsine square root of the proportion (VC/TL) or as log_10_(1/6+AN).

### Aphid survival and intrinsic growth rate

To measure aphid survival, a cohort of five apterous adults was placed on a trifoliate leaf of a 3-week-old plant and caged. Sixteen hours after infestation, adult aphids were removed and 10 nymphs were left per caged leaf. Nymph survivorship was determined daily until they reached adulthood after 6 d. The experiment was set in a completely randomized design in the glasshouse with 10 replicates per accession.

To assess the intrinsic growth rate, an apterous SAA adult (p1) was caged on the adaxial surface of the youngest, fully expanded trifoliate leaf of a 3-week-old plant and allowed to deposit nymphs. When the reproduction of p1 began, the first nymph produced was moved to a different leaf of the same plant and caged separately. The nymphs produced by each female were counted over the following 4 d and removed after each 24h period to determine the days to first progeny (*d*) and the total number of progeny (*M*d). The intrinsic rate of natural increase (*r*_m_) of the *T. trifolii* population was calculated using a modification of the Wyatt and White equation, to determine the effect of the plant genotypes on SAA population growth potential (Wyatt and White, 1977):


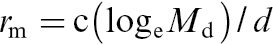
(1)

where *d* is the length of the pre-reproduction period (days to first progenies), assuming aphid reproduction is constant during the estimated reproductive day, *M*_d_ is the mean number of larvae born during the observation period (4 d) multiplied by the estimated reproductive period (*d*), and *c*=0.738, the constant index for an aphid species. Data were analysed following transformation for aphid survival (arcsine square root of the proportion of surviving aphids) or untransformed for the *r*_m_ data. The experiment was set in a completely randomized design in the glasshouse with 10 replicates per accession.

### Host selection behaviour

Three-week-old plants, grown in separate 0.45 l pots in a growth chamber, were placed in insect-proof cages. The boxes were covered with fine, light-transmitting mesh on the top and on three sides with a sliding Perspex cover on the remaining side. Each box (blocks; *n*=10) contained one plant per genotype equidistant from a platform (5cm diameter Petri dish) suspended in the centre of the cage, ~10cm above the base of the cage. SAA alatae were released onto this platform. The pots were spaced so that the plants did not touch each other. Twenty SAA alatae were placed on the platform in each cage and allowed to choose host plants on which to feed and reproduce over the next 48h. Settling of aphids on each plant was observed at 3, 6, 12, 24, and 48h after release. The proportion of aphids per plant was analysed as the arcsine square root of the proportion.

### Aphid feeding behaviour

The feeding behaviour of SAAs on 3-week-old plants of Jester, A17, and A20 was studied using the direct-current EPG technique (Tjallingii, 1988) as described by Klingler *et al.* (2005) with modifications. A single apterous adult SAA was placed on a single trifoliate leaf and feeding behaviour was monitored. Twenty biological replicates were included for each accession (Jester, A17, and A20). A six-channel amplifier simultaneously recorded six individual aphids on separate plants, two Jester, two A17, and two A20 plants per day for 10 d. Waveform patterns in this study were scored according to the categories described by Tjallingii and Esch (1993).

### Genetic analysis of SAA resistance

#### Hybrids and biparental populations

F_1_ hybrids derived from reciprocal crosses between A17 and A20 were confirmed as true hybrids by PCR using the simple sequence repeat markers 002B07 and 001G03 (http://medicago.org/genome/downloads.php, last accessed on 2 September 2013). The RILs population was generated by random single seed descent from F_2_ individuals derived from two F_1_ hybrids derived from crosses where A20 was the pollen donor.

#### Experimental design and biometrics

The RILs, F_1_ hybrids, F_2_ individuals, and parental lines were evaluated in a randomized complete block design. Three weeks after sowing, six blocks were infested, placing two apterous adults per plant. From each block, data was collected from one individual per RIL, one to three F_1_ hybrid plants per cross type, and two to five plants per parental accession. Aphid migration within the block was not restricted. One day before infection, the number of leaves per plant was counted and the length of the largest leaf was measured to estimate the shoot fresh weight (eSFW-0) based on the best linear fit derived from weighing 10 F_2_ individuals representing the populations’ variation in shoot weight:


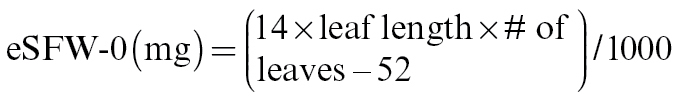


The response to aphid infestation was assessed at 7 and 21 dpi by quantifying the degree of aphid population build-up (aphid movement and reproduction; AN-7, AN/TL-7, ADW-21, and ADW/PDW-21), the VC symptoms (VC-7, VC/TL-7, VC-21, and VC/GL-21), or the degree of plant damage (DL/TL-21, SDW-21, SFW-21 and lnSFW-21) (see Table 1 for abbreviations). ANOVA was performed using a general mixed model with parental lines/hybrids as fixed effects and blocks as random effect, or using a model with two random factors: RILs and blocks. Pearson’s correlation indices (Supplementary Table S1 at *JXB* online) were determined to evaluate the association between traits.

**Table 1.** Response of A17, A20, and F_1_ hybrids derived from reciprocal crosses to SAA infestation

Trait	Abbreviation (transformation)^a^	Mean^*a*^				*F* ^*b*^	*R* ^*2b*^
		A17	F_1_ (A20×A17)	F_1_ (A17×A20)	A20		
Estimated plant fresh weight before infestation (mg)	eSFW-0 (sqR)	0.67	0.73	0.67	0.56	0.3811	0.050
Number of aphids per plant at 7 dpi	AN-7 (log)	0.80 a	6.80 b	12.50 b	12.7 b	<0.0001	0.491
Ratio of number of aphids by number of leaves per plant at 7 dpi	AN/TL-7 (log)	0.05 a	0.44 ab	0.68 b	0.77 b	<0.0001	0.415
Number of leaves with VC at 7 dpi	VC-7 (log)	1.00	0.00	0.31	0.59	0.3522	0.000
Proportion of plants showing VC at 7 dpi	VC/TL-7 (arcsine)	0.04	0.00	0.02	0.04	0.4148	0.000
Number of leaves with VC at 21 dpi	VC-21 (log)	0.00 a	3.80 b	5.60 b	8.50 c	<0.0001	0.805
Proportion of green leaves per plant with VC at 21dpi	VC/GL-21 (arcsine)	0.00 a	0.09 b	0.17 b	0.41 c	<0.0001	0.678
Proportion of dead leaves per plant at 21 dpi	DL/TL-21 (arcsine)	0.16 a	0.15 ab	0.23 ab	0.33 b	<0.0001	0.412
Shoot dry weight at 21 dpi (mg)	SDW-21	0.87	0.94	0.93	0.90	0.8735	0.088
Shoot fresh weight at 21 dpi (g)	SFW-21	5.00	7.20	5.90	5.10	0.0586	0.188
Fresh weight increment through infestation (mg)	InSFW-21 (log)	7.60 a	6.50 ab	5.30 ab	10.4 b	0.0109	0.225
Aphid dry weight per plant at 21 dpi (mg)	ADW-21 (log)	5.70 a	7.70 ab	11.7 bc	14.0 c	<0.0001	0.555
Ratio of aphid (mg) by plant (g) dry weight at 21 dpi	ADW/PDW-21 (log)	6.70 a	8.40 ab	13.4 bc	15.5 c	<0.0001	0.435

^*a*^ Means are presented in non-transformed values followed by SD. ANOVA and mean comparisons were done on transformed data: sqR, square root; log, natural log, log e; arcsine, inverse sine square root of proportion; otherwise, no transformation was applied to the data. For each trait, different letters indicate significantly different means.

^*b*^ Probability of *F* and *R* ^*2*^ values obtained from ANOVA including the blocks in the model (REML analysis); the percentage of the random variance explained by the blocks was low (0.0–6.4%; not shown).

#### Genotyping and linkage analysis

The RILs were genotyped at F_6_ (*n*=114) using a set of 89 simple sequence repeat markers distributed evenly throughout genome as described by Guo *et al.* (2012). Details of the RIL genotypes and the resulting linkage map have been described previously by Guo *et al.* (2012). The genetic map spans 445.3 cM with an average distance of 4.79 cM between markers.

#### QTL analyses

RILs were phenotyped at F_7_ (*n*=92). QTL detection and characterization was performed using MultiQTL v2.6 (http://www.MultiQTL.com, last accessed on 2 September 2013) with the general interval mapping and marker restoration options for a RIL-selfing population. Residual heterozygosity was treated as missing data. The hypothesis that a single locus had an effect on a single quantitative trait was evaluated as described by Lichtenzveig *et al.* (2006).

## Results

### Resistance response to aphid infestation

**Fig. 1.**
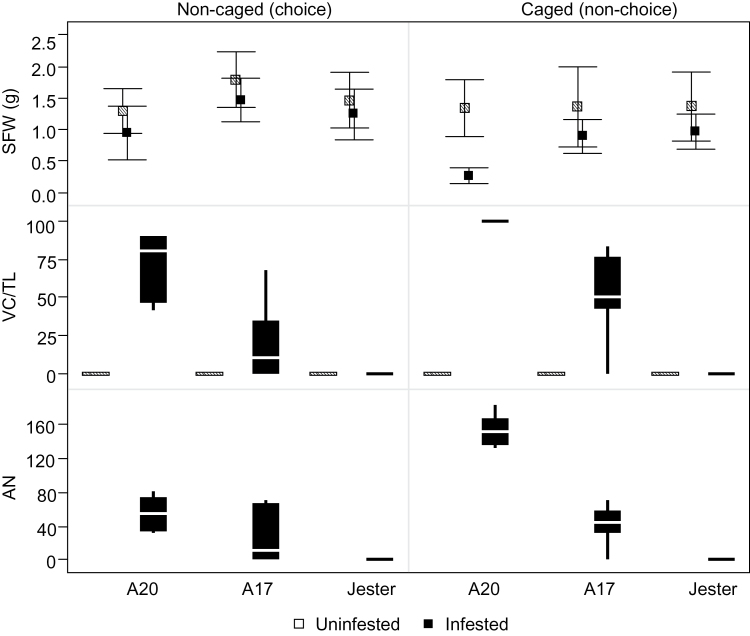
*M. truncatula* response to SAA infestation measured as SFW, as the proportion of leaves showing VC (VC/TL), or total AN per plant at 17 dpi. SFW values are represented by means (black, infested; hatched, non-infested controls); error bars are 95% confidence intervals around the mean. VC/TL and AN values are represented by outlier box plots (the box represents the distribution of half of the observations, the horizontal white line is the median, and the whiskers are the lower and upper quartiles). For each experiment there were six to eight experimental units.

Previous studies have shown that *M. truncatula* harbours varying degrees of resistance to SAA (Gao *et al.*, 2007*b*). High levels of resistance were observed in cultivars Jester, Cyprus, and Caliph with all nymphs dying within 24–48h. Comparatively, A17 and Mogul were less resistant (40–80% SAA mortality rates), but were significantly more resistant than the highly susceptible accessions Borung and A20. In the above experiments, aphids were restricted to a single plant and the aphid developmental stage was disregarded. Here, we focused on the two closely related lines Jester and A17, and a third accession, A20, and evaluated the response to SAA infestation in depth using choice and non-choice experiments (Fig. 1). In the choice experiment, aphids were free to move within the infested plots; in the non-choice experiment, the plants were individually caged, restricting the aphids’ movement between plants.

**Fig. 2.**
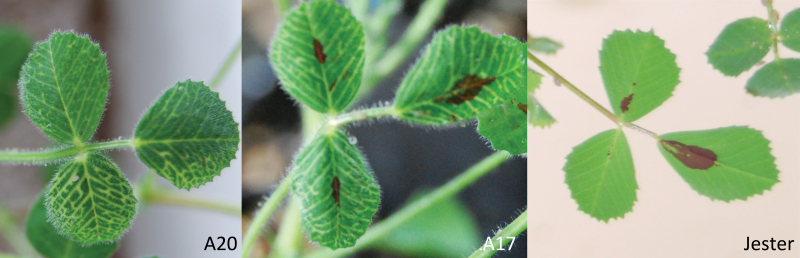
Systemic VC in leaves approximately orthostichous to infested leaves of *M. truncatula* accessions in A20, A17, and Jester.

In the choice experiment, accessions had a similar SFW irrespective of whether they were infested with SAA (*F*_Acc×Treat_=0.9320; |*t*|>0.1505). Substantial differences between accessions were observed in AN and in the incidence and severity of systemic VC (Figs 1 and 2). Aphids were observed in all A20 plants and in only half of the A17 plants (four out of eight); no living aphids were observed on Jester plants at 17 dpi. The AN per infested plant in A20 was not significantly different from that on A17 plants (|*t*|=0.9941; AN mean=54.9, *n=*8, and AN mean=56.5, *n*=4, for A20 and A17, respectively). The colonization level also correlated with the incidence of VC. While all A20 plants had a high proportion of VC leaves per plant (VC/TL; Fig. 1), only the A17 plants infested with aphids (four out of eight) were affected, and the VC/TL in these plants was significantly lower than in infested A20 plants (|*t*|=0.0365; VC/TL mean=0.78, *n=*8, and VC/TL mean=0.38, *n*=4, for A20 and A17, respectively). No VC was observed in Jester (Fig. 2). More dramatic differences were observed in the non-choice experiment. At 17 dpi, the SFW of infested A20 plants was significantly lower compared with uninfested plants and the other accessions (|*t*|=0.0012; SFW mean=1.35g, *n=*6, and SFW mean=0.28g, *n*=8, for uninfested and infested, respectively; Tukey–Kramer HSD test *F*<0.05). No significant differences in SFW were observed between A17 and Jester (Fig. 1). Similar to the choice experiment, the accessions differed in AN and in the incidence and severity of VC. However, when aphid movement was restricted, A17 had a higher VC incidence (seven out of eight plants were affected) and severity (VC/TL mean=52.1). No changes were observed in Jester. In both experiments, most aphids were found on the adaxial side of leaves.

### Dissection of resistance response

#### Tolerance

**Fig. 3.**
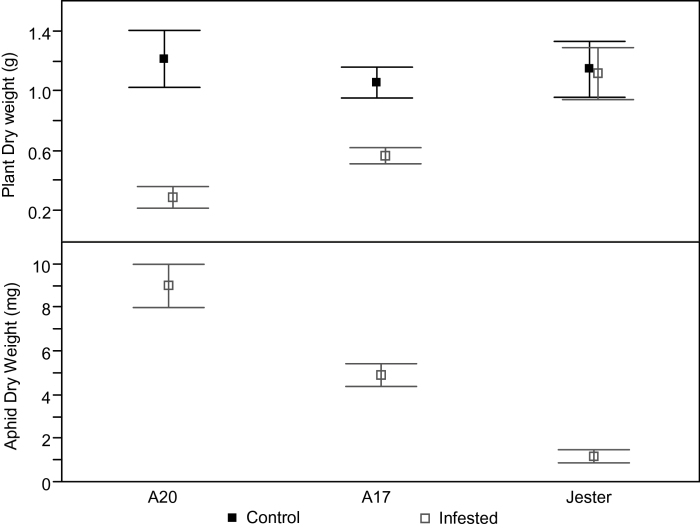
*M. truncatula* accessions differ in their response to SAA infestation measured as SDW and on their effect on total ADW per plant at 21 dpi. Plants were each infested with 50 adult aphids in a complete randomized non-choice experiment. For each experiment, *n*=10. Full-factorial ANOVA resulted in a significant interaction between treatment and accession (*F*_Acc×Treat_<0.0001, *df*=2, *R* ^2^=0.771). SDW values are represented by means (square symbols); error bars are 95% confidence intervals around the mean.

The response of the *M. truncatula* accessions was evaluated under higher infestation pressure in a non-choice experiment to assess tolerance. Three-week-old plants were infested with 50 SAA adults each, in a completely randomized non-choice experiment (whole plants caged). The number of aphids per plant was maintained as quasi-constant by removing or adding aphids every 3 d for 21 d. Under these conditions, shoot dry weight (SDW) in both A20 and A17 differed significantly between infested and uninfested plants (/*t*/<0.0001). No significant differences in SDW were observed between the uninfested accessions (*F*=0.3223). The SDW of infested A17 plants was significantly higher than that of A20 but significantly lower than that of Jester (Tukey–Kramer HSD test, *F*<0.05), suggesting that A17 is partially tolerant to SAA (Fig. 3).

#### Antibiosis

**Fig. 4.**
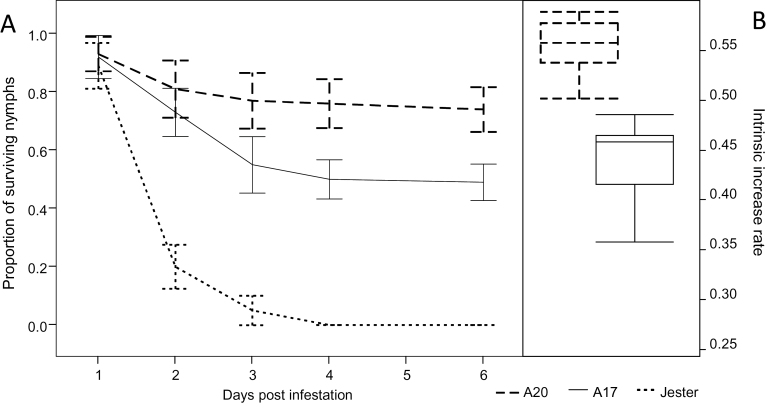
Effect of *M. truncatula* accessions on nymph survival and intrinsic rate of increase. (A) The proportion of survival on A20, A17, and Jester was monitored from first-instar nymph to adult stage in a non-choice experiment; each plant was infested with five cohort nymphs. Error bars are 95% confidence intervals of the mean (*n*=10). (B) The intrinsic rate of increase (*r*_m_) of aphids was calculated based on pre-reproductive time in days and number of offspring produced on single caged leaves per plant of A17 and A20 (*n*=10); no aphid reproduction was observed on Jester. Data are represented by outlier box plots.

The accessions were compared in terms of nymph survivorship (Fig. 4). No significant differences in nymph survivorship were observed between accessions at 1 dpi (Prob *F* =0.4398), most likely because *T. trifolii* can survive for 24h without feeding (Gao *et al.*, 2007*b*). The percentage of surviving nymphs at 2 dpi was significantly higher on A20 and to a lesser extent on A17 compared with Jester (*F<*0.0001; *R* ^*2*^=0.83). Only a few aphids survived on the highly resistant plant Jester at 3 dpi, by 4 dpi all nymphs had died, and no adults were found on Jester at 6 dpi. Nymph survivorship on A17 was also significantly lower than on A20 from 3 dpi onwards (*F<*0.0001; *R* ^*2*^=0.86). From this stage, aphids had settled on their respective host plants (either A17 or A20) and all survivor nymphs had reached adulthood (Fig. 4A).

The intrinsic rate of increase (*r*_*m*_) is another reliable parameter to estimate antibiosis resistance to insects by measuring insect nymph reproduction and days to first progeny on susceptible and resistant plants (Wyatt and White, 1977). Even though no significant differences were detected between nymphs reared on A17 and A20 in the days to first progeny (*d*; |*t*|*=*0.0746) or total progeny (*M*_d_; |*t*|=0.0593), highly significant differences were observed in their respective *r*_*m*_ values (*F<*0.0001, *R* ^2^=0.65). As expected, no aphid reproduction was observed on Jester, which is reflected in the *r*_m_ value of zero (Fig. 4B).

#### Antixenosis

**Fig. 5.**
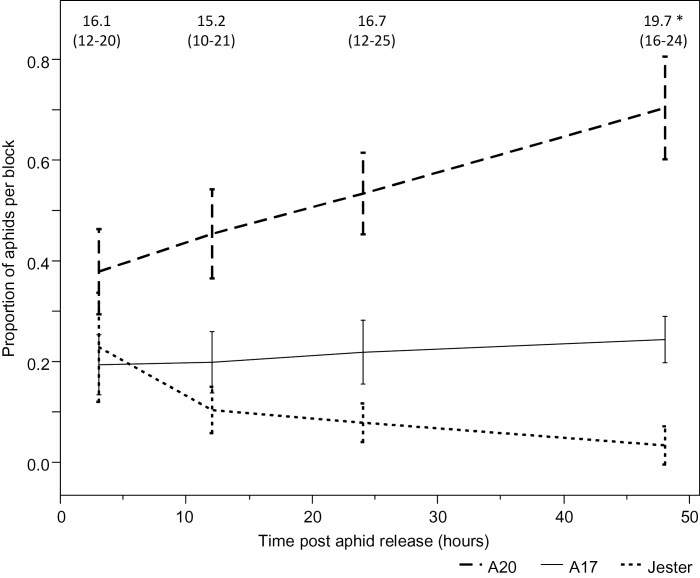
The settling of SAA alatae in a host choice experiment on *M. truncatula* accessions. Error bars are 95% confidence intervals of the mean (*n*=10). The mean (minimum–maximum) number of aphids per block is indicated.

Upon release of the aphids on the platform, the alatae quickly dispersed to a host plant. The mean number of alatae that settled on A17 remained relatively constant over the 48h experimental period (Fig. 5; *b*=0.0015; *t*_*b*_=0.0973, *R* ^*2*^=0.07), but significantly decreased on Jester and increased on A20 (*b*=0.08; *t*_*b*_<0.0001, *R* ^*2*^=0.47; Fig. 5). At 3h post-release (hpr), SAA preferred to settle on A20 over A17 or Jester (*F*<0.05). As early as 12 hpr, the accessions differed relative to the number of aphids per plant (*F*<0.05).

### Resistance is expressed in the phloem

**Fig. 6.**
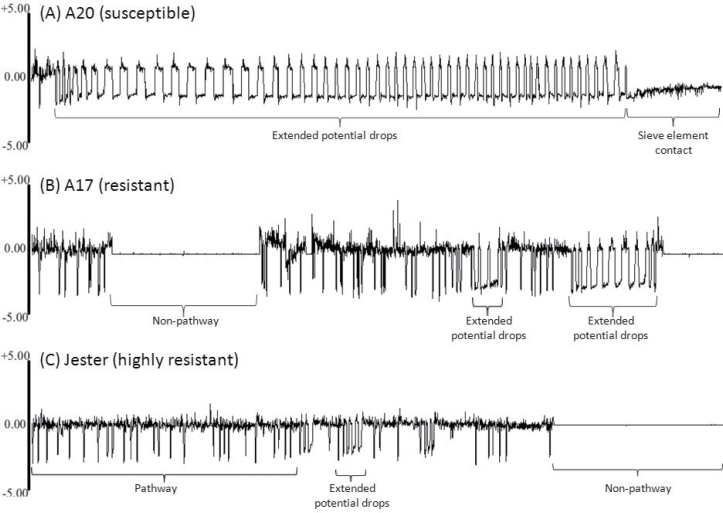

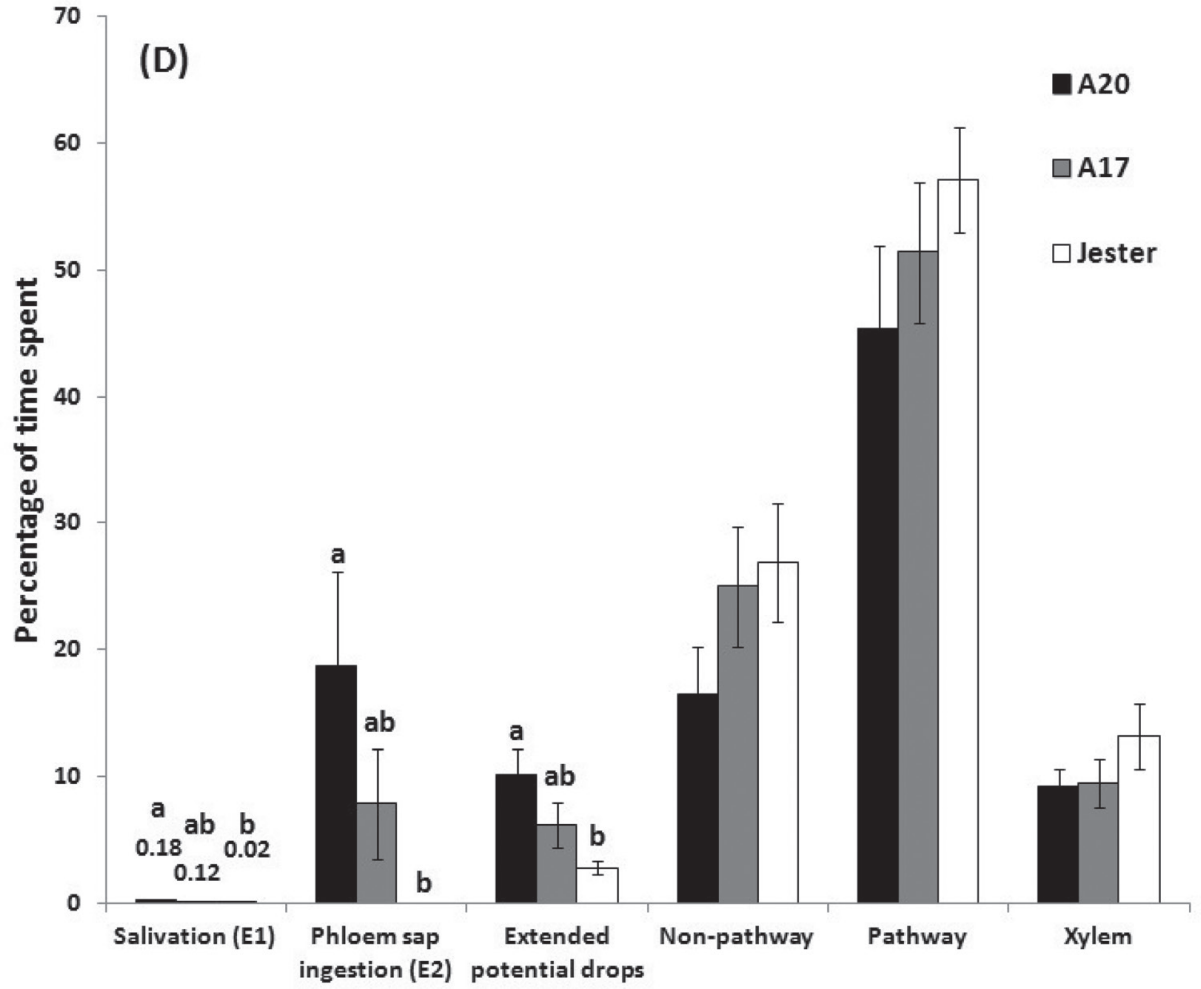

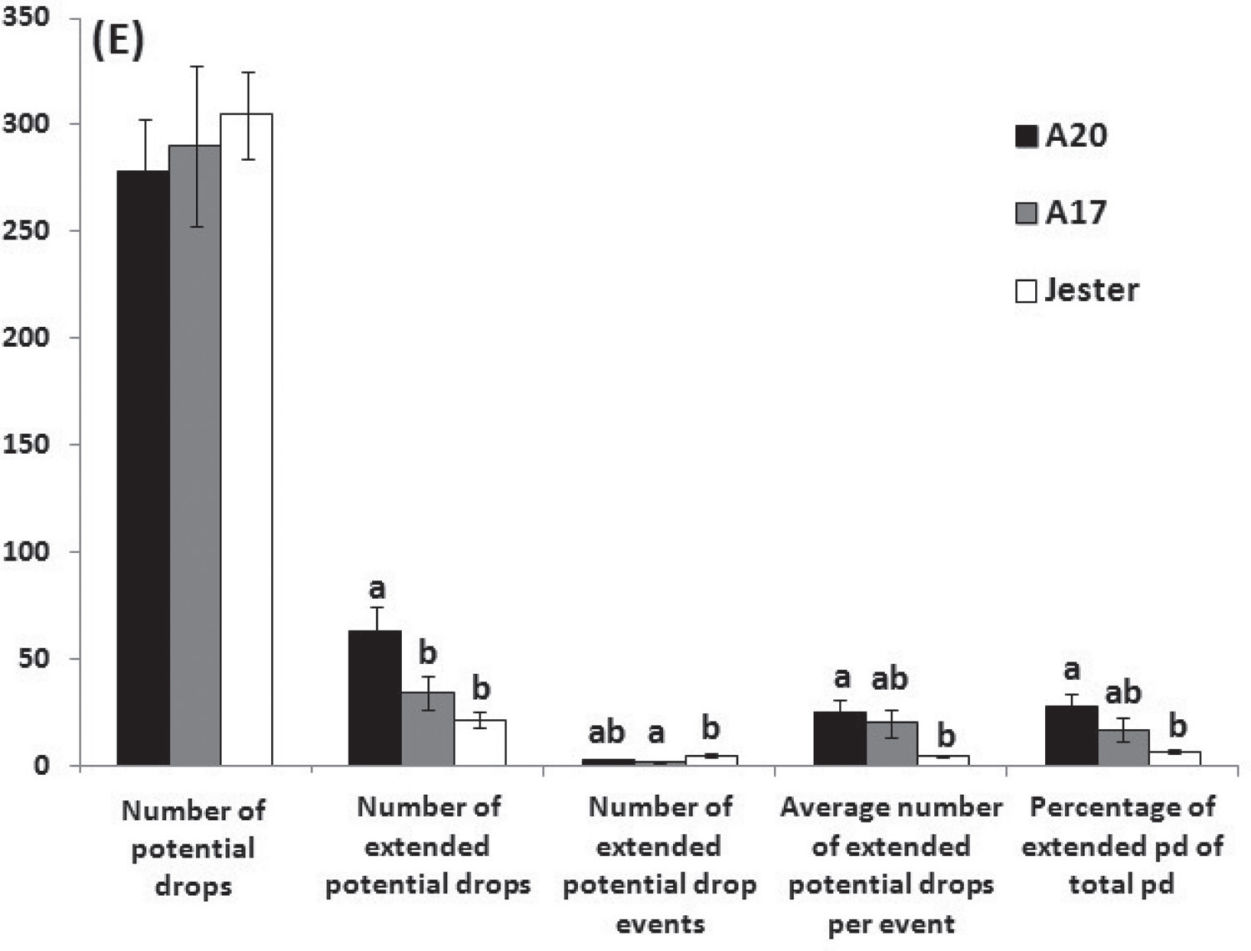
(A–C) EPG showing representative waveform patterns produced when SAA apterae feed on the susceptible accession A20 (A), moderately resistant accession A17 (B), or highly resistant accession Jester (C). The horizontal axes represent a 1h time period; the vertical axes represent voltage. Histological studies of plant–aphid interactions have correlated stylet positions in plant tissues with specific EPG waveforms (Tjallingii, 1988). ‘Non-pathway’ indicates stylets are outside the plant, while ‘pathway’ indicates mostly intramural probing activities between mesophyll or parenchyma cells. ‘Sieve element contact’, consisting primarily of sap ingestion with short periods of salivation into sieve elements, was frequently seen with plants of A20 and significantly less on plants of A17 and only rarely seen with plants of Jester. Sharp, downward spikes, named potential drops, indicate cell puncture events by stylets, each lasting approximately 5 s. A novel waveform that we termed extended potential drops were sharp downward spikes that lasted more than 5 s. (D) Percentage of time SAA apterae spent in various activities on A20, A17 and Jester during 9h exposure to the host plants. (E) Detailed analysis of the two different types of potential drops in the three *M. truncatula* lines. Each value represents the mean±SEM of 12–14 biological replicates. Means for the accession not connected by the same letter are significantly different (*P*<0.01).

The EPG technique is a powerful method to observe in real time the locations and activities of aphid stylets during probing, including their salivation into sieve elements and passive uptake of phloem sap (Tjallingii, 1978; Tjallingii and Esch, 1993). Representative waveforms are presented in Fig. 6A–C. The accessions did not differ significantly in the proportion of time that the apterae spent outside the cuticle (non-probing), penetrating between cells *en route* to the vascular tissue (pathway phase), or contacting xylem, or in the number of potential drops (Fig. 6D, E).

In contrast to events prior to successful puncturing of the phloem sieve element, the proportion of time spent by SAA secreting saliva in the sieve element (E1 phase) and the subsequent ingestion of phloem sap (E2 phase) were significantly reduced for SAA feeding on Jester compared with the susceptible A20 plants, while A17 had an intermediate phenotype between these two accessions. The secretion of saliva on A20 plants occupied an average of 0.18% of the total recorded activity, compared with 0.12% on A17 and 0.02% on Jester plants. Similarly, the ingestion of phloem sap on A20 plants averaged 18.7%, compared with 7.8%, while no phloem sap ingestion occurred on Jester (Fig. 6D). Of the 12–14 replicates tested for each line, 58% of A20 plants had phloem sieve element contact, compared with 42% of A17. The reduction in salivation into the sieve element and phloem sap ingestion in contrast to all the other (pre-feeding) activities measured suggests that resistance to SAA in both Jester and A17 is phloem mediated.

A novel waveform was observed during the 9h recordings following SAA infestation of A20 and A17 (Fig. 6A, B). Sharp potential drops with downward spikes indicative of cell puncture events typically lasted ~5 s each. When conducting EPG experiments with SAA on the three *M. truncatula* lines, the sharp downward spikes lasted longer, 10–37 s, and therefore were referred to as ‘extended potential drops’ (Fig. 6A, B). These extended potential drops always occurred in quick succession with as few as three extended potential drops per event and as many 81. The average number of extended potential drops was significantly more on A20 plants (63.3) compared with A17 (34.3) and Jester (21.4; Fig. 6E) plants. Interestingly, the lowest number of potential drop events was observed on A17 plants with an average of 2.1 per recording, while A20 had on average 3.1 per recording and Jester 4.9. This resulted in a significantly higher average number of per repeated extended potential drop events for the susceptible A20 (25.2) compared with the resistant Jester (4.5), with A17 showing an intermediate phenotype with 20.0 extended potential drops per event (*F*<0.05). Phloem sap ingestion (E2) occurred only after repeated extended potential drops; the repeated extended potential drop events of SAA adults on A20 plants were observed in 58% of recordings compared with 42% of recordings for A17.

### Novel QTLs associated with the interaction of *M. truncatula* with SAAs

**Fig. 7.**
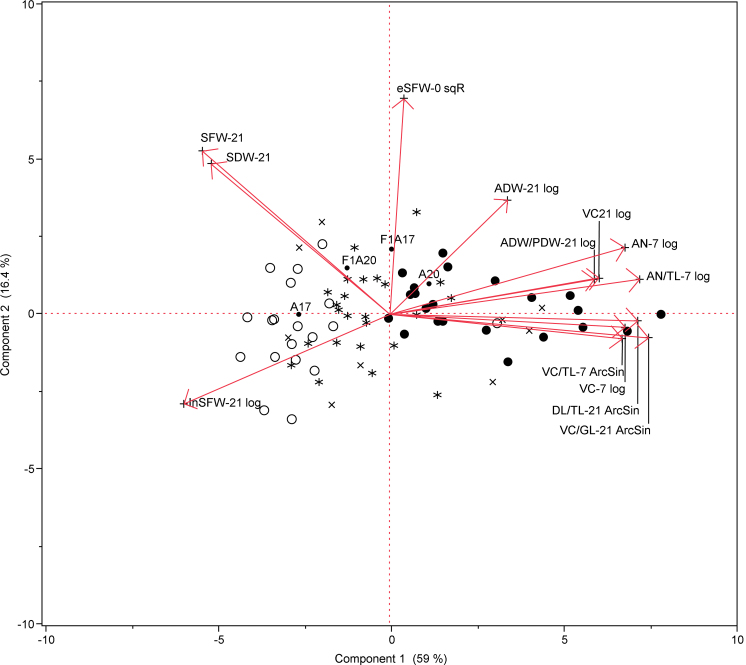
Principal component analysis biplot representing the overall response to SAA infestation in A17, A20, the F_1_ hybrids, and RILs derived from A17×A20 crosses. The arrows (loadings) represent the variables measured in response to SAA infestation (calculated from the line means); the arrow lengths represents the variance of the variable, and the angles between the arrows represents the correlation between the variables. The scores represent the line means. The scores for parental lines and F_1_ hybrids are shown by a tagged dot; the RIL scores are labelled according to their genotype at the h2_151m16a (LG3) and h2_2p16d (LG) loci, respectively: *AAAA* (cross), *AABB* (open circle), *BBAA* (closed circle), *BBBB* (star). RILs heterozygous at either of the loci were not included in the graph. (This figure is available in colour at *JXB* online.)

The genetic basis of interactions between *M. truncatula* and SAAs was studied using F_1_ hybrids (derived from reciprocal crosses between A17 and A20) and F_2:7_ RILs infested with two adult aphids per plant. To account for potential differences in plant growth independent of infestation, plant measurements were taken prior to infestation to determine eSFW-0; the parental lines and F_1_s did not differ significantly in their eSFW-0 values (Table 1). The overall response of the host plants was presented in a principal component biplot (Fig. 7) and the correlation indexes between traits based on the line means in Supplementary Table S1. Principal components one and two explained 75.4% of the total variation in the responses to SAA infestation (Fig. 7). There was no significant correlation between eSFW-0 and variables associated with disease, except the variables describing growth at 7 and 21 dpi (SWF-21, SDW-21, and InSFW-21; Fig. 7 and Supplementary Table S1). Following infestation, measurements of aphid population build-up and plant damage symptoms were recorded at 7 and 21 dpi (Table 1). Higher numbers of aphids were observed on A20 compared with A17 at 7 dpi, while the F_1_ hybrids behaved similarly to A20 (Table 1). Based on the results described in previous sections, these responses are probably due to the effect of antibiosis. At 7 dpi, only a few leaves had started to show VC, but no differences were observed between genotypes. However, there was a strong correlation between the number of aphids per plant at 7 dpi (AN-7 or AN/TL-7) and the occurrence of VC at 21 dpi (Fig. 7 and Supplementary Table S1). At 21 dpi, A20 had a higher proportion of dead leaves and leaves with VC than A17; the F_1_ hybrids showed an intermediate response (Table 1 and Fig. 7). Higher numbers of aphids were observed on A20 as shown by the aphid dry weight (ADW) per plant (Table 1). No significant differences were observed between parental accessions and the F_1_ hybrids in their final shoot dry and fresh weight (SDW-21 or SFW-21). Plant weight at 21 dpi was inversely correlated to the number of aphids per plant and the proportion of dead leaves or VC leaves at 7 or 21 dpi (Fig. 7 and Supplementary Table S1).

**Fig. 8.**
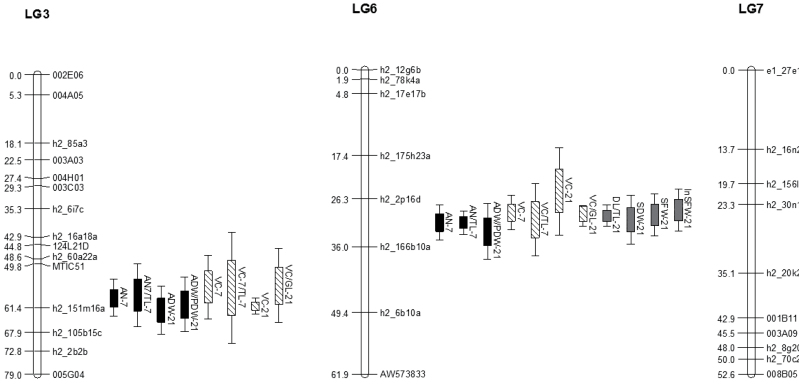
Genetic map position of the QTLs involved in SAA resistance based on phenotype data for the A17×A20 RIL population. Interval distances are given in cM. The genomic locations of the QTLs are depicted by bars on the right side of linkage groups, with SD depicted by whiskers on either side of the bar. Solid bars of the QTLs are associated with antibiosis (e.g. aphid numbers), hatched bars with VC, and grey bars with tolerance (e.g. plant growth).

QTL analysis revealed three loci associated with the *M. truncatula* interaction with SAAs, one on linkage group 3 (LG3), one on LG6, and one on LG7 (Fig. 8 and Table 2). The locus in LG3, flanked by the markers MTIC51 and h2_105b15 and spanning ~18 cM (interval 11–12; Table 2), had significant effects on variables describing AN and the occurrence of VC leaves at 7 and 21 dpi (Fig. 8 and Table 2). The ‘resistance’ allele, associated with reduced aphid numbers and less VC, originated from A17. This locus had no effect on plant-related measurements other than VC (i.e. the effect on DL/TL-21, SW-21, or InSFW-21 was not significantly different from zero; Table 2). The second locus, in LG6 flanked by the markers h2_175h23a and h2_166b10a and spanning ~23 cM, had significant effects on all the variables related to aphid infestation: AN, appearance of VC, plant damage,e and biomass (Fig. 8 and Table 2). In this case, the ‘resistance’ allele, associated with reduced aphid numbers, less VC, and larger plants was derived from A20 (Table 2). The third locus on LG7 was associated with AN at 21 dpi (Fig. 8 and Supplementary Table S2 at *JXB* online). The effect of this locus was 1.75 and 1.85mg g^–1^ for ADW-21 and ADW/PDW-21, respectively. Positive effect values for these traits indicated that the allele associated with the reduction of aphids was derived from A20. At 21 dpi, aphids started to move from the most susceptible plants to healthier plants, which might explain the effect of the LG7 locus. A locus in LG8 was associated with the estimated weight of seedlings prior to infestation (eSFW-0); this locus was not linked to any of the loci associated with the response to infestation (Supplementary Table S2).

**Table 2.** Features of QTLs associated with M. truncatula response to SAA in the A17×A20 RIL populationThe results were obtained from assuming a 1qtl-1trait genetic model.

		**LG3**								**LG6**							
		LOD^*a*^	Prob^*b*^	Int.^*c*^	Dist. (SD)^*d*^	PEV^*e*^	Effect^*f*^	Mean^*g*^	Model^*h*^	LOD	Prob	Int.	Dist, (SD)	PEV	Effect	Mean	Model
Aphid numbers	AN-7	5.93	<0.0001	11	58.8 (4.9)	0.296	–4.4 aphids	3.0 aphids	G	7.04	<0.0001	5	31.1 (3.6)	0.324	4.3 aphids	3.7 aphids	D
	AN/TL-7	4.64	<0.0001	12	58.1 (8.3)	0.232	–1.9 aphids	0.5 aphids	D	9.47	<0.0001	5	31.1 (2.4)	0.331	2.3 aphids	0.5 aphids	G
	ADW-21	4.75	0.0004	12	62.1 (6.4)	0.250	–1.8 mg	6.0 mg	D	2.06	0.0920						
	ADW/ PDW21	4.23	0.0004	12	60.6 (7.2)	0.217	–1.8mg/g	9.4mg/g	D	4.59	0.0006	5	32.9 (5.7)	0.242	1.9mg/g	10.2mg/g	D
Vein chlorosis	VC-7	4.61	<0.0001	11	56.0 (8.4)	0.254	–1.5 leaves	1.4 leaves	D	4.87	<0.0001	5	29.0 (3.5)	0.229	1.4 leaves	1.5 leaves	D
	VC/TL-7	10.57	0.0136	11	56.2(14.6)	0.485	–0.008	0.006	G	12.2	0.0002	5	30.5 (7.4)	0.156	0.012	0.008	G
	VC-21	10.46	<0.0001	12	61.0 (2.2)	0.464	–1.8 leaves	2.8 leaves	D	2.64	0.0164	4	24.7 (8.9)	0.136	1.3 leaves	3.1 leaves	D
	VC/GL-21	3.68	0.0022	11	55.6 (9.7)	0.191	–0.106	0.207	D	12.8	<0.0001	5	29.7 (2.1)	0.284	0.475	0.249	G
Plant growth/ tolerance	DL/TL-21	1.53	0.0892							15.0	<0.0001	5	29.7 (2.2)	0.348	0.259	0.309	G
	SDW-21	0.74	0.5004							3.37	0.0040	5	30.5 (5.0)	0.174	–0.16 mg	0.74 mg	G
	SFW-21	0.68	0.6118							3.42	0.0008	5	29.5 (4.3)	0.166	–0.96 g	4.52 g	G
	InSFW-21	0.56	0.4364							7.65	<0.0001	5	28.5 (4.3)	0.325	–1.62	7.24	G

^*a*^ Calculated using a permutation test (5000 permutations).

^*b*^ Significance test H1: Linkage Group (LG) significantly different from zero on the variable. QTL analyses were done using transformed data. Models with Prob > 0.05 were disregarded.

^*c*^ Interval in the LG most probably associated with the quantitative trait.

^*d*^ Distance in cM.

^*e*^ The proportion of phenotypic variance explained by the genetic model.

^*f*^ QTL effect on the trait/variable. The phenotypic difference between A17-like and A20-like allelic groups is presented. Effects are expressed as non-transformed values.

^*g*^ Mean response of the RIL population expressed as non-transformed values.

^*h*^ Submodel that is most likely to fit the data (*P*>0.05). D, default: assumes no ‘variance’ effect (Korol *et al.*, 1996); G, general: allows for the allelic groups to differ in variance.

## Discussion

In this study, we characterized the moderate resistance to SAA in the *M. truncatula* accession A17, compared with the highly resistant Jester and the susceptible A20, and dissected the genetic basis of the moderate resistance in A17. Resistance to aphid attack in A17 could have been caused by antibiosis, antixenosis, or tolerance, or any combination of these. These different aspects of aphid resistance were thus investigated. The non-choice assays on both nymphs and SAA adults showed that antibiosis is a major component of the resistance phenotype in A17 (Figs 1 and 4) compared with the susceptible A20. Similarly, a moderate resistance in A17 to both BGA and the Australian PA biotype compared with Jester and A20 was identified by Klingler *et al.* (2009) and Guo *et al.* (2012). In contrast to these moderate levels of resistance, the antibiosis resistance of A17 to the European PA biotype PS01 is much stronger, causing a negative mean relative growth rate of the aphid on A17, with lethality to aphids as early as 48h after infestation (Stewart *et al.*, 2009). Therefore, there appear to be varying degrees of antibiosis resistance to different aphids present in the *M. truncatula* reference accession A17.

The systemic VC caused by SAA infestation in A20 and to a lesser degree in A17 is a hallmark of damage by SAA in *M. truncatula* (Klingler *et al.*, 2007). In alfalfa, this damage was shown to be caused by the aphid *per se* rather than an aphid-borne virus (Paschke and Sylvester, 1957; Nickel and Sylvester, 1959). Consistent with the report by Klingler *et al.* (2007), in the present study VC in both A20 and A17 was initiated exclusively on still-expanding, systemic leaves approximately orthostichous to the infested leaf. The occurrence of systemic VC was significantly more frequent on A20 plants compared with A17 plants in both choice and non-choice experiments and was not observed on any Jester plants (Fig. 1). Systemic VC is probably a host response to a bioactive component(s) or derivative(s) of SAA saliva; however, further research is needed to confirm this.

In the non-choice experiments, the SFW of infested and uninfested A20 plants differed significantly, indicating a lack of tolerance. To estimate better the degree of tolerance in A17 compared with the highly susceptible A20 and highly resistant Jester, high SAA infestation pressure was applied, and these conditions showed that SDW in both A20 and A17 differed significantly between infested and uninfested plants (Fig. 3). The SDW of infested A17 plants was significantly higher than that of A20 but significantly lower than that of Jester, suggesting that A17 is partially tolerant to SAA (Fig. 3). Partial tolerance in A17 to BGA and PA has been identified previously, and this tolerance to both aphid species was controlled by distinct loci (Klingler *et al.*, 2009; Guo *et al.*, 2012).

Host selection by alatae is regarded as the first stage of colonization and therefore plays a major role in aphid establishment in the field. In repeated host choice experiments with alatae of SAA, the alatae preferentially settled on the susceptible accession A20, with alatae rapidly migrating from Jester to A20 plants, leading to significant differences in AN as early as 12 hpr (Fig. 5). Aphid numbers on A17 over the course of the experiment remained relatively unchanged (Fig. 5), indicating that antixenosis in A17 does not play a major role in the resistance phenotype, particularly when compared with the resistance observed in Jester. Antixenosis does not play a significant role in the moderate resistance phenotype observed in A17 to PA, where no significant differences were observed in settling behaviour between A17 and the susceptible A20 (Guo *et al.*, 2012). This is in contrast to observations for both BGA and PA choice experiments between Jester and A17, where a significant difference in settling behaviour was observed at 6 and 24h after alatae release for BGA and PA, respectively (Klingler *et al.*, 2005; Gao *et al.*, 2008).

In the EPG studies using adult SAAs, a reduction in salivation into the sieve element and phloem sap ingestion on resistant Jester compared with susceptible A20 was identified (Fig. 6D). Aphids on A17 also showed a significant reduction in phloem sap ingestion compared with aphids on A20, and showed significantly less sustained phloem sap ingestion (sustained E2 waveform longer than 10min) than on A20; 10min is a threshold often used as an indicator of phloem acceptance (Tjallingii, 2006). A longer duration of E1 salivation with a shorter duration of phloem sap ingestion generally suggests that aphids secrete more saliva into the sieve elements to counter a plant defence response such as the plugging of sieve pores (Will and Van Bel, 2006; Will *et al.*, 2007, 2009), whereas here the duration of salivation and phloem sap ingestion were both longest on susceptible A20 plants. Deterrent compound(s) present in the phloem sap of A17 could explain the difference in sustained phloem sap ingestion between A17 and A20. Alternatively, frequent extracellular salivation (E1e) following watery salivation (E1) in A17 generally indicates cell collapse (Tjallingii, 2006), which could be a symptom of cell death induced by recognition of effectors in the saliva of SAA. This hypothesis is supported by the presence of localized chlorotic flecks surrounding the feeding site of SAAs, as observed in highly resistant lines such as Jester and Mogul (Klingler *et al.*, 2007); such damage was not observed in A17. Whatever the cause, the frequent disruptions of salivation in A17 resulted in SAAs spending significantly less time ingesting phloem sap, which in turn is consistent with the relatively lower growth rate of SAAs in both caged leaf and whole plant assays on A17 compared with A20 (Figs 1 and 3).

Another interesting finding is the novel waveform of extended potential drops, which often occurred in rapid succession, with the highest average number of sequential extended potential drops on susceptible A20 plants (Fig. 6E). Why SAAs repeatedly use these extended potential drops is unclear. The frequency of extended potential drops was shortest when SAAs were placed on highly resistant Jester plants, but the number of extended potential drop events was the highest on Jester. Furthermore, in all cases of phloem sap ingestion (E2), this waveform was preceded by a series of extended potential drops. The extended potential drops could thus be a specialized mechanism that SAAs adopted to determine whether they can or cannot establish a successful feeding site. Further research is needed to determine whether the observed extended potential drops are unique to the interaction between SAAs and *M. truncatula* or whether it is observed in SAAs interacting with other legumes, as no other EPG studies have been conducted with SAAs to date. Taken together, these results, in contrast to all the other (pre-feeding) activities measured, suggest that resistance to SAA in Jester and A17 is phloem mediated. Similar experiments conducted in *M. truncatula* lines resistant and susceptible to BGA, PA, and cowpea aphid revealed significant reductions in phloem feeding on resistant lines. Therefore, all identified aphid resistance traits in *M. truncatula* to date appear to be exerted through the phloem (Klingler *et al.*, 2005; Gao *et al.*, 2008; Guo *et al.*, 2012; Kamphuis *et al.*, 2012).

The genetic dissection of SAA resistance in the RIL population derived from A17 and A20 identified intervals on LG3, LG6, and LG7 involved in different aspects of the plant interaction with SAAs (Figs 7 and 8, and Table 2). The interval on LG3 between markers MTIC51 and h2_105b15c spans a region rich in classical coiled-coiled nucleotide-binding site leucine-rich-repeat (CC-NBS-LRR) resistance genes and is several centiMorgans distal to the major SAA resistance gene, *TTR*, present in the *M. truncatula* cultivars Mogul and Jester (Klingler *et al.*, 2007). This region harbours other aphid resistance genes previously identified, including the major dominant resistance genes to the Australian PA biotype termed *APR* identified in *M. truncatula* accession A17 (Gao *et al.*, 2008; Guo *et al.*, 2009) and to the European PA biotype LS01 termed *RAP1* (Stewart *et al.*, 2009), and a quantitative locus for reduction in plant dry weight following PA infestation in A17 (Guo *et al.*, 2012). The interval on LG6 between markers h2_175h23a and h2_166b10a harbours at least 20 open reading frames similar to NBS-LRR resistance genes and three open reading frames similar to LRR-receptor-like serine/threonine protein kinase resistance genes. However, this interval has several gaps in the assembly of the reference *M. truncatula* A17 genome (GBrowse Mt3.5; http://www.medicagohapmap.org/?genome, last accessed on 2 September 2013). A region distal to this interval on LG6 in A17 harbours a moderate resistance locus termed *RAP2* involved in the resistance response to the European PA biotype LS01 (Stewart *et al.*, 2009). The ‘resistance’ allele on LG3 is derived from A17 and results in a lower aphid biomass at 7 and 21 dpi as well as less VC, whereas the ‘resistance’ allele on LG6 is derived from A20, and has a large effect on plant tolerance, as well as AN and VC. The ‘resistance’ allele on LG3 explains the clear antibiosis effect observed in A17, whereas the ‘resistance’ allele on LG6 explains the temporary tolerance observed in A20 during the infestation, where the biomass of the plant is relatively unaffected and the A20 plants temporarily tolerate large aphid densities, until they succumb to the aphid infestation by being drained of nutrients. If these quantitative resistance loci on LG3 and LG6 are in fact similar in sequence homology to ‘classical’ resistance genes, it raises the question of how these *R* genes are able to mediate and control such specific aspects of the resistance (e.g. antibiosis or tolerance) and also why they are less effective compared with the strong-acting single dominant resistance *TTR* gene in *M. truncatula* cv. Jester.

The region identified on LG7 plays a role in antibiosis resistance at 21 dpi, and this region has not previously been associated with a response to aphid infestation in *M. truncatula*. This seems to be a late-acting QTL compared with the other two as it is only involved in antibiosis at 21 dpi, when the aphids have started to migrate from the most susceptible plants to healthier plants (Supplementary Table S2). It has no role in the early response to SAA infestation or plant tolerance and therefore does not play as significant a role in SAA-mediated resistance compared with the QTLs identified on LG3 and LG6. It thus appears that some loci for SAA resistance and resistance to aphid species of the genus *Acyrthosiphon* cluster together on LG3 and LG6 in regions rich in *R* gene-like sequences. This is also observed in another legume, soybean, where genes for aphid resistance to various soybean aphid biotypes also cluster together on chromosomes 7 (LG M) and 13 (LG F) (Zhang *et al.*, 2009; Jun *et al.*, 2012). In contrast a resistance locus to foxglove aphid is located on chromosome 3 (Ohnishi *et al.*, 2012).

To date, moderate resistance to three different aphid species in A17 has been identified and in all three cases antibiosis is the major contributor to the moderate resistance phenotype, where resistance is phloem mediated. Furthermore, varying degrees of tolerance were observed and antixenosis does not play a major role in these moderate resistance phenotypes. This is in contrast to the biology of resistance in *M. truncatula* to cowpea aphid, controlled by a major QTL, and resistance controlled by the major dominant resistance genes *AKR*, *APR*, and *TTR* to BGA, PA, and SAA, respectively, in *M. truncatula* as these involve a combination of antibiosis, antixenosis, and tolerance (Klingler *et al.*, 2005; Gao *et al.*, 2008; Kamphuis *et al.*, 2012). In soybean, different loci control various aspects of resistance to soybean aphid. Major resistance genes to soybean aphid such as *Rag1* involve a combination of antibiosis and antixenosis (Hill *et al.*, 2006), whereas QTLs *qRa_1* and *qRa_2* only involve antibiosis and not antixenosis (Meng *et al.*, 2011), as observed for the moderate SAA resistance loci identified herein.

Over the last decade, there has been considerable progress in understanding plant–aphid interactions at a number of levels and in a range of plant species, including *Arabidopsis*, tomato, and two prominent legume species: *M. truncatula* and soybean (Kamphuis *et al.*, 2013). These studies have provided insight into the genetics and molecular mechanisms underlying resistance to generalist and specialist aphids. Efforts are also being made to gain a better understanding of how the aphid manipulates its plant host to establish successful feeding sites (Hogenhout and Bos, 2011). This has mainly been made possible by efforts of the International Aphid Genome Consortium, who sequenced the first aphid genome of PA, along with the wealth of growing genomic/genetic resources on both sides of key plant–aphid interactions (IAGC, 2010). The identification and characterization of these novel resistance loci to SAA further add to the potential of the *M. truncatula* model for studying plant defence against sap-sucking insects. These advances may also lead to a greater understanding of the key determinants in the plant for successful resistance and in the aphid for successful infestation. From this increased understanding, there should emerge strategies to better control these economically damaging pests.

## Supplementary data

Supplementary data are available at *JXB* online.

Supplementary Table S1. Genetic correlation indexes for traits describing the *M. truncatula* response to spotted alfalfa aphid.

Supplementary Table S2. Features of QTLs associated with *M. truncatula* response to spotted alfalfa aphid (SAA) in the A17×A20 recombinant inbred line (RIL) population.

Supplementary Data

## Abbreviations:

ADW: aphid dry weight
AN: aphid number
BGA: bluegreen aphid
dpi: days post-infestation
EPG: electrical penetration graph
eSFW: estimated shoot fresh weight
hpr: hours post-release
LG: linkage group
LRR: leucine-rich-repeat
NBS: nucleotide-binding site
PA: pea aphid
QTL: quantitative trait locus
RIL: recombinant inbred line
SAA: spotted alfalfa aphid
SDW: shoot dry weight
SFW: shoot fresh weight
TL: total number of leaves
VC: vein chlorosis.
