# Supplementary material for: Characterization and genetic dissection of resistance to spotted alfalfa aphid (Therioaphis trifolii) in Medicago truncatula
Source: J Exp Bot. 2013 Sep 21;64(16):5157–72. doi: 10.1093/jxb/ert305 (PMC3830491; doi:10.1093/jxb/ert305)
Supplement: Supplementary Data [file supp_64_16_5157__index.html]

Characterization and genetic dissection of resistance to spotted alfalfa aphid (Therioaphis trifolii) in Medicago truncatula — Characterization and genetic dissection of resistance to spotted alfalfa aphid (Therioaphis trifolii) in Medicago truncatula — Supplementary Data 

# Characterization and genetic dissection of resistance to spotted alfalfa aphid (*Therioaphis trifolii*) in *Medicago truncatula*

## Supplementary Data

Data files

**Files in this Data Supplement:**

- Supplementary Data - Supplementary Data
