# Supplementary material for: Characterization and genetic dissection of resistance to spotted alfalfa aphid (Therioaphis trifolii) in Medicago truncatula
Source: J Exp Bot. 2013 Sep 21;64(16):5157–72. doi: 10.1093/jxb/ert305 (PMC3830491; doi:10.1093/jxb/ert305)
Supplement: Supplementary Data [file supp_ert305_jexbot106310_file001.pdf]

# Characterisation and genetic dissection of resistance to spotted alfalfa aphid (*Therioaphis trifolii*) in *Medicago truncatula*

*Lars G Kamphuis, Judith Lichtenzveig, Kefan Peng, Su-min Guo, John P Klingler, Kadambot H M Siddique, Lingling Gao, and Karam B Singh*

**Table S1.** Genetic correlation indexes for traits describing the *M. truncatula* response to spotted alfalfa aphid

| Trait | Abbreviation (transformation)a | AN-7 (log) | AN/TL-7 (log) | DL/TL-21 (ArcSin) | SDW-21 | SFW-21 | InSFW-21 (log) | ADW-21 (log) | ADW/PDW-21 (log) | VC-7 (log) | VC-21 (log) | VC/TL-7 (ArcSin) | VC/GL-21 (ArcSin) |
|---|---|---|---|---|---|---|---|---|---|---|---|---|---|
| Estimated plant fresh weight before infestation | eSFW-0 (sqR) | 0.27 ** | 0.16 | 0.09 | 0.41 *** | 0.53 *** | -0.56 *** | 0.17 | -0.02 | -0.01 | 0.13 | -0.06 | -0.01 |
| Total number of leaves per plant at 7 dpi | AN-7 (log) | | 0.96 *** | 0.75 *** | -0.41 *** | -0.38 *** | -0.70 *** | 0.44 *** | 0.62 *** | 0.66 *** | 0.75 *** | 0.64 *** | 0.78 *** |
| Number of aphids per plant 7 dpi | AN/TL-7 (log) | | | 0.81 *** | -0.50 *** | -0.50 *** | -0.72 *** | 0.44 *** | 0.66 *** | 0.73 *** | 0.72 *** | 0.72 *** | 0.84 *** |
| Proportion of dead leaves per plant at 21 dpi | DL/TL-21 (ArcSin) | | | | -0.59 *** | -0.64 *** | -0.75 *** | 0.33 ** | 0.62 *** | 0.70 *** | 0.59 *** | 0.70 *** | 0.89 *** |
| Plant dry weight at 21 dpi | SDW-21 | | | | | 0.90 *** | 0.31 ** | 0.03 | -0.44 *** | -0.48 *** | -0.43 *** | -0.50 *** | -0.66 *** |
| Plant fresh weight at 21 dpi | SFW-21 | | | | | | 0.32 ** | -0.04 | -0.45 *** | -0.56 *** | -0.40 *** | -0.58 *** | -0.68 *** |
| Fresh weight increment through infestation | InSFW-21 (log) | | | | | | | -0.21 * | -0.38 ** | -0.61 *** | -0.53 *** | -0.59 *** | -0.70 *** |
| Aphid dry weight (mg) per plant at 21 dpi | ADW-21 (log) | | | | | | | | 0.87 *** | 0.35 ** | 0.38 ** | 0.30 ** | 0.34 ** |
| Ratio of aphid (mg) by plant (g) dry weight at 21dpi | ADW/PDW-21 (log) | | | | | | | | | 0.57 *** | 0.53 *** | 0.55 *** | 0.65 *** |
| Proportion of plants showing VC at 7 dpi | VC-7 (log) | | | | | | | | | | 0.54 *** | 0.99 *** | 0.78 *** |
| Proportion of green leaves with vein chlorosis per plant at 7 dpi | VC-21 (log) | | | | | | | | | | | 0.50 *** | 0.79 *** |
| Number of leaves with vein chlorosis at 21dpi | VC/TL-7 (ArcSin) | | | | | | | | | | | | 0.78 *** |
| Proportion of green leaves with vein chlorosis per plant at 21dpi | VC/GL-21 (ArcSin) | | | | | | | | | | | | |

a) Mathematical transformations: sqR = square root; log = log10; ArcSin = inverse sine square root of proportion; otherwise, no transformation was applied to the data

b) Probability of *r* (correlation index): * 0.05 > Prob. > 0.01; ** 0.0099 < Prob. < 0.0001; *** Prob. < 0.0001. Tests included the mean response of RILs, F1 and parental lines ($n$ = 92-96)

**Table S2.** Features of QTLs associated with *M. truncatula* response to spotted alfalfa aphid (SAA) in the A17 x A20 recombinant inbred line (RIL) population. The results were obtained from assuming a 1qtl-1trait genetic model.

| | ADW-21 | ADW/PDW-21 | eSFW-0 |
|---|---|---|---|
| | LG7 | | LG8 |
| LOD[a] | 4.2 | 4.07 | 3.382 |
| Prob [b] | 0.0006 | 0.001 | 0.0034 |
| LG-interval[c] | 7 | 6 | 1 |
| Distance (SD) [d] | 48.2 (4.3) | 45.2 (5.0) | 3.1 (9.5) |
| PEV[e] | 0.222 | 0.222 | 0.1629 |
| Effect[f] | 1.75 mg | 1.85 mg/g | 0.01 g |
| Mean[g] | 6.05 mg | 9.39 mg/g | 0.54 g |
| Genetic Model[h] | Default | Default | Default |

[a] Calculated using a permutation test (5000 permutations).
[b] Significance test H1: Linkage Group (LG) is significantly different from zero on the variable. Transformed data was used in the QTL analysis. Models with Prob > 0.05 were disregarded.
[c] Interval in the LG most likely associated with the quantitative trait.
[d] Distance in cM.
[e] The proportion of phenotypic variance explained by the genetic model.
[f] QTL effect on the trait/variable. The phenotypic difference between A17-like and A20-like allelic groups is presented. Effects are expressed as non-transformed values.
[g] Mean response of the RIL population expressed as non-transformed values.
[h] Submodel that is most likely to fit the data (Prob > 0.05); Default, assumes no “variance” effect (Korol *et al.*, 1996), General, allows for the allelic groups to differ in variance.
